# Finding gene clusters for a replicated time course study

**DOI:** 10.1186/1756-0500-7-60

**Published:** 2014-01-24

**Authors:** Li-Xuan Qin, Linda Breeden, Steven G Self

**Affiliations:** 1 Department of Epidemiology and Biostatistics, Memorial Sloan-Kettering Cancer Center, New York, NY 10065, USA; 2 Division of Basic Science, Fred Hutchinson Cancer Research Center, Seattle, WA 98109, USA; 3 Division of Public Health Science, Fred Hutchinson Cancer Research Center, Seattle, WA 98109, USA

**Keywords:** Clustering, Microarray, Regression, Replications, Time course

## Abstract

**Background:**

Finding genes that share similar expression patterns across samples is an important question that is frequently asked in high-throughput microarray studies. Traditional clustering algorithms such as K-means clustering and hierarchical clustering base gene clustering directly on the observed measurements and do not take into account the specific experimental design under which the microarray data were collected. A new model-based clustering method, the clustering of regression models method, takes into account the specific design of the microarray study and bases the clustering on how genes are related to sample covariates. It can find useful gene clusters for studies from complicated study designs such as replicated time course studies.

**Findings:**

In this paper, we applied the clustering of regression models method to data from a time course study of yeast on two genotypes, wild type and *YOX1* mutant, each with two technical replicates, and compared the clustering results with K-means clustering. We identified gene clusters that have similar expression patterns in wild type yeast, two of which were missed by K-means clustering. We further identified gene clusters whose expression patterns were changed in *YOX1* mutant yeast compared to wild type yeast.

**Conclusions:**

The clustering of regression models method can be a valuable tool for identifying genes that are coordinately transcribed by a common mechanism.

## Findings

### Background

Clustering is a useful tool to look for unknown groupings of objects [1]. It has become an important part of the analysis of gene expression data, owing to the pioneering work of Eisen et al. [2]. By looking for clusters of genes that have similar expression levels across samples, researchers hope to better understand gene functions, genetic pathways, and regulatory circuits. Cluster analysis can also be used to cluster samples; we will focus on the problem of gene clustering in this paper.

A number of analytic methods have been applied to the problem of gene clustering. They can largely be classified to two categories: (1) algorithmic clustering methods, such as K-means clustering and hierarchical clustering [2,3]; and (2) model-based clustering methods, such as the multivariate normal mixture model [4,5]. These methods generally do not take into account the experimental design, such as cross-sectional (CS) design, longitudinal with no replication (LNR) design, and longitudinal with replications (LWR) design.

We previously developed a model-based clustering method, the clustering of regression models (CORM) method, which employs regression to model gene expression and clusters genes based on their relationship between expression levels and sample covariates [6]. Different from previous clustering methods, CORM partitions systematic variation from non-systematic variation and bases the clustering on systematic variation only. CORM is applicable to data collected under various designs for microarray experiments and takes into account the specific experimental design in the modeling. We have previously implemented the Clustering of Linear Models (CLM) method and the Clustering of Linear Mixed Models (CLMM) method, and applied them to analyze data collected under the CS design and the LNR design, respectively.

Our contributions in this paper are as follows: (1) we illustrate the methodologic advantages of the CORM method over K-means clustering, (2) we demonstrate the application of the CLMM method to gene expression data collected under the LWR design, using a yeast time course dataset measured for two yeast cell lines each with two technical replicates, and (3) we show empirical evidence of CLMM’s benefits compared to K-means through a comparison of the clustering results for the yeast data – two clusters were uniquely identified by CLMM but missed by K-means and a spurious cluster was picked up by K-means and spared by CLMM.

### Methods

#### ***K-means clustering***

Given a set of objects, K-means clustering seeks a partition of all objects into K groups to minimize the total within group sum of squared Euclidean distance [7]. The minimum could, in theory, be found by searching over all possible clusterings; however, this approach is computationally prohibitive when the number of objects is large. An iterative procedure is instead adopted to search for the minimum. Specifically, K-means starts with an initial value for the cluster centers, then iterates between the cluster-assigning step (each object is assigned to the closest cluster center) and the cluster-center-recalculating step (each cluster center is updated as the average of objects assigned to that cluster), until convergence.

It has been pointed out that K-means is equivalent to assuming a multivariate normal mixture model with component distributions having the same scalar covariance matrix and equal mixture proportions, and then fitting the model using an EM algorithm to maximize the classification likelihood [8-10]. Here notation is introduced for LWR data, including CS data and LNR data as special cases. Let **y**_gi_ denote the vector of expression levels for gene g and sample i, **y**_g_ = (**y**^T^_g1_, . . . , **y**^T^_gm_)^T^ the vector of expression levels for gene g for sample 1 through sample m, G the number of genes, and K the number of clusters. Let u_g_ denote the cluster membership for gene g. The model underlying K-means can be written as

$$y_{g}|\;\left(u_{g}=k\right)=\mu_{k}+\epsilon_{g}$$

$$\epsilon_{g}\sim \mathrm{MVN}\left(0,\sigma^{2}I\right)$$

where **ϵ**_g_ is the vector of measurement errors, **I** is an identity matrix, and u_g_ is a random variable on (1, 2, . . . , K) with probabilities π_k_ = 1/K. Cluster memberships are considered as missing data in the EM algorithm: cluster-assigning step corresponds to the E-step and cluster-center-recalculating step to the M-step.

#### ***The CORM method***

For the problem of differential expression analysis, the regression modeling framework has been employed to characterize systematic variation in the expression profile of each gene and distinguish it from random variation. Differential expression is identified by contrasting expression levels measured under different experimental conditions or by identifying dependencies on concomitantly measured covariates. The resulting estimated regression models can provide an accurate and precise description of expression profiles. Similarly, the regression model framework can be used for the problem of gene clustering: systematic variation is separated from random variation and gene clustering is based solely on the systematic part of the variation. We call this the clustering of regression models method (CORM) [6].

Let **X**_gi_ (n_gi_ × p) denote the design matrix for gene g and sample i, **F**_**β**k,**ξ**k_ the conditional distribution of genes in cluster k given the covariates with parameters **β**_k_ and **ξ**_k_, **β**_k_ (p × 1) the vector of regression coefficients, and μ(.; .) the regression function. The model underlying CORM can be written as

$$y_{\mathrm{gi}}|\;\left(X_{\mathrm{gi}},u_{g}=k\right)\sim {F_{\beta }}_{k,\xi k}$$

$$E\left(y_{\mathrm{gi}}|X_{\mathrm{gi}},u_{g}=k\right)=\mu \left(X_{\mathrm{gi}};\beta_{k}\right)$$

where u_g_ is a random variable on (1, 2, . . . ,K) with probabilities (π_1_, π_2_, . . . , π_K_). Complete specification of the CORM modeling framework requires identification of the error structure (parameterized by **ξ**), which depends on the form of the regression model. The specific form of the regression model used for CORM is flexible. For example, it can be the linear model, the linear mixed model, the nonlinear model, and the nonparametric regression model. Its choice should depend on the experimental design and the scientific question. The EM algorithm can be used to fit the CORM model [11,12]. Implementation details can be found in [6] for the clustering of linear models (CLM) method and the clustering of linear mixed models (CLMM) method.

#### ***Comparing K-means and CORM***

**Figure 1:**
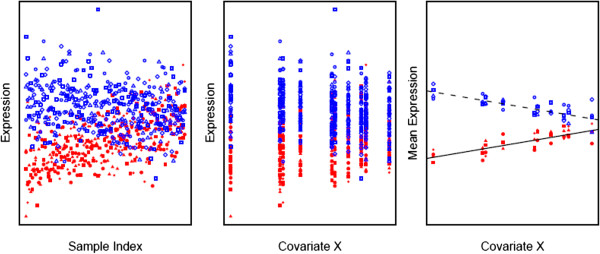
**Compare K-means and CLM. Data is simulated for eight genes and 100 samples.** Eight genes belong to two clusters. Each of the ten levels of covariate X has ten samples. Left panel plots gene expression *versus* sample index. Middle panel plots gene expression *versus* covariate X. Right panel plots average gene expression for samples at the same X level *versus* covariate X. Symbols represent genes and colors represent gene clusters.

Both K-means and CORM are partitional clustering methods (as opposed to hierarchical clustering methods), which concern the problem of the optimal partitioning of a given set of objects into a prespecified number of mutually exclusive and exhaustive clusters. However, the two methods base clustering on different features of a gene. The feature of interest for K-means is the vector of sample-specific expectations for a gene. For each sample-specific expectation, sample size is 1 and genes in the same cluster are used as replicates for its estimation. K-means does not make any assumption on the relationship between the expected expression level and the covariates and is ‘model-free’ in this respect. The feature of interest for CORM is the vector of regression parameters shared by samples for a gene. It separates systematic variation from random variation and increases clustering precision especially when the sample size is large (Figure 1). Note that although CORM is ‘model-based’ in terms of modeling expression levels with covariates, the regression model itself can be either parametric or nonparametric (for example, use of spline basis for modeling longitudinal data).

The different gene features considered by K-means and CORM also have implications on other important issues of cluster analysis. We will comment on three such issues here, one with impact before gene clustering and two after gene clustering.

(a) Selection of genes. A microarray provides measurements on thousands of genes, but it is common to select a small subset (tens to hundreds) of genes to cluster, especially for partitional clustering. One reason to select a subset is to keep the computation manageable. Another reason is to try to exclude uninformative genes to prevent them from deteriorating the clustering. For K-means, however, ‘uninformative’ is not well defined. One might select the most variable genes. However, that strategy does not distinguish genes with large signal and genes with large noise when including genes; nor does it distinguish genes with small signal and genes with small noise when excluding genes. With CORM, informative genes can be selected using a per-gene regression model and a significance cutoff appropriately adjusted for multiplicity [13], and genes with significant systematic variation are clustered to find those that are similarly associated to the covariates. CORM and regression-based differential expression analysis can thus form an integrated framework for the analysis of microarray data.

(b) Characterization and interpretation of clusters. After clustering genes, it is useful to determine the cluster signatures for the identified clusters. Often they are set to be the cluster centers. CORM clusters can be identified by their regression coefficients and have a specific interpretation depending on the experimental design. For example, we can tell whether a gene cluster tends to be up-regulated or down-regulated comparing diseased samples to normal samples. The interpretability of CORM clusters allows a more interpretable comparison of gene clusters identified in different data sets with similar experimental designs – not only the clustering of genes can be compared but also the characteristics of the clusters.

(c) Application of clusters. The average of genes in the same cluster has been proposed to act as predictors for sample classification [14]. CORM tends to find clusters that are more stable across samples, as we will show later. In addition, CORM provides an explicit prediction rule for new genes that are measured on a new set of samples.

CORM provides an alternative clustering method for scenarios when K-means has limitations. For example, while applicable to both CS data and LNR data, K-means does not distinguish the two experimental designs. K-means cannot naturally handle LWR data – profiles of a gene need to be averaged or connected first. K-means might not use all information in the data; for example, in a longitudinal study, it considers time points to be exchangeable and ignores their ordering and correlation. Unlike K-means, CORM can naturally deal with missing values for any gene or sample (under the assumption of missing at random) as well as imbalanced experimental design (for example, different sampling times for different samples in a longitudinal study). Moreover, CORM can easily incorporate technical replicates together with biological replicates in a hierarchical manner.

The gains of CORM depend on the truth of the regression model and its robustness to model misspecification. Ideally, the design of an experiment determines the gene-related feature available for clustering and hence informs parameterization of the regression model for CORM. Experimental design should be chosen to produce the feature that most likely reflects biological clusters of interest. For example, a longitudinal design can be used to find clusters of genes that behave similarly across time, while a cross-sectional design can be used to find clusters of genes that behave similarly across different levels of covariate (for example, disease stage).

### Results

To study the regulation of the cell cycle in yeast, we studied gene expression across the cell cycle for both wild type (WT) yeast and single mutant (SM) yeast with the *YOX1* gene knocked out [15]. Alpha factor was used for cell synchronization and 6,227 ORFs were measured at 5-min intervals for 120 min. cDNA microarrays were used with a common reference mRNA obtained from logarithmically growing wild type yeast cells and log ratios were used to measure expression levels. Replicate measurements were obtained for both WT yeast and SM yeast. Our goal was to identify co-expression behavior of cell cycle dependent genes. Using three microarray data sets across the yeast cell cycle published by [16], Zhao et al. (2001) identified a set of 256 genes whose transcription is cell cycle dependent in at least two out of the three data sets using a per-gene regression modeling approach [17]. We focused on these 256 periodic genes in our analysis.

The primary goal of our analysis is to cluster genes that have similar expression patterns among WT yeast. As a secondary goal, we clustered genes using both WT yeast and SM yeast to identify genes whose expression patterns are changed by the mutation. Unlike K-means clustering, CLMM can explicitly accommodate both the replication and the sample covariate (mutation status). In addition, CLMM can naturally deal with the imbalanced experimental design: WT had one bad time point at 105 min and SM had three at 25 min, 40 min, and 55 min, due to technical problems (for example, sample loss, poor hybridization). These bad time points were removed from the cluster analysis. There was also missing data: 41 measures belonging to 17 genes for WT data and 17 measures belonging to 17 genes for SM data were clearly outliers based on signal strength compared to other time points, which arise due to technical failures of the measurement procedure rather than reflecting true biological variation that should be modeled. See Additional file 1.

#### ***Cluster WT data***

**Figure 2:**
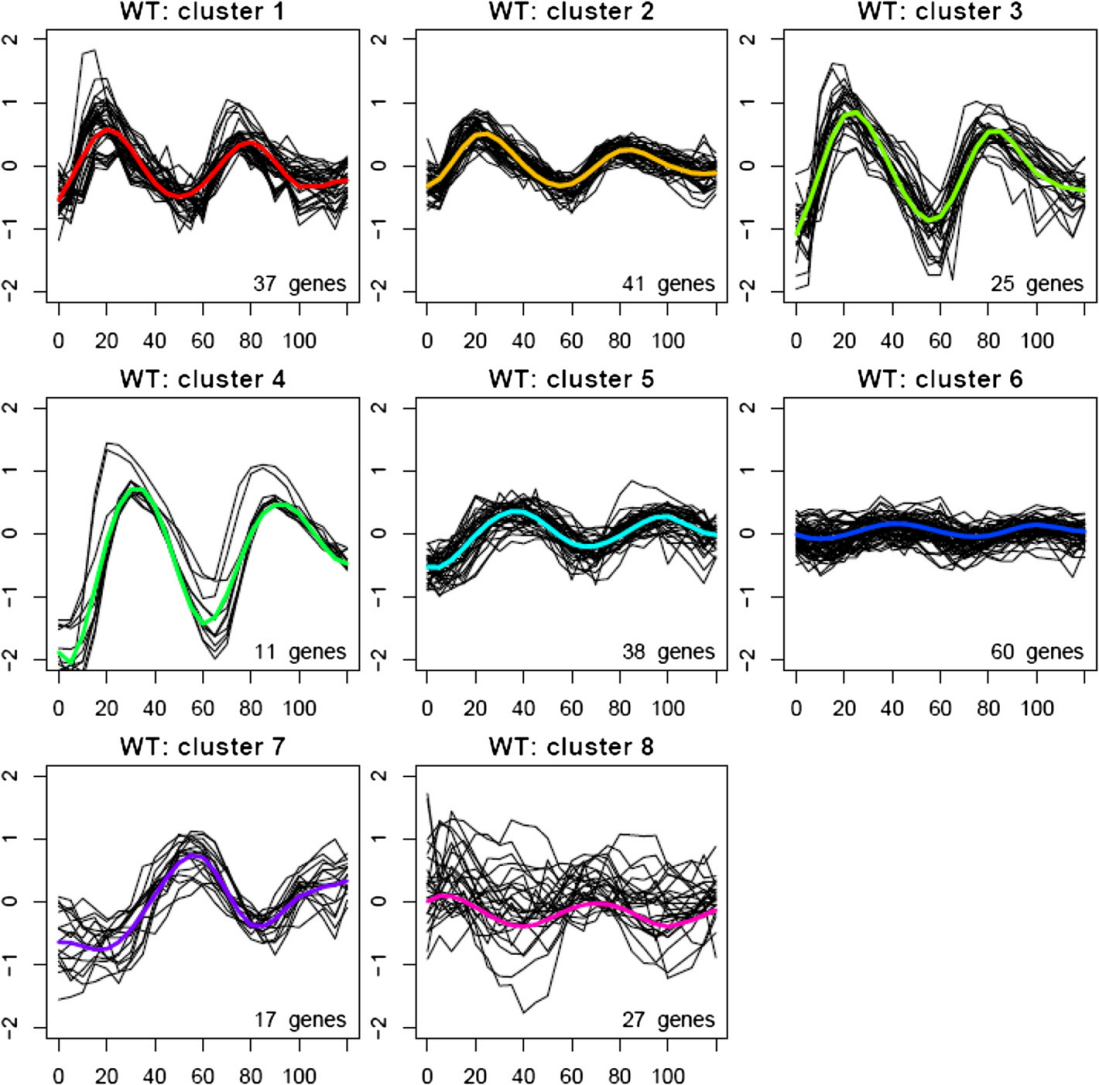
**Clustering of the 256 genes based on the CLMM method using the WT data.** Genes were clustered to eight clusters. Each panel plots the fitted profile (colored line) of one cluster and the observed profiles (black line) of genes in that cluster averaged across the two WT samples *versus* time in minutes. The number of genes in each fitted cluster is labeled at the lower right corner of each panel.

CLMM was applied to cluster the 256 genes using WT data. The design matrix for fixed effects was the B-spline basis for time 0-120 min with 7 equally spaced knots. The number of knots was set to be 7 to allow a flexible modeling of the expression profiles and at the same time to avoid overfitting. Within a reasonable range, the clustering results were not sensitive to the number of knots for the B-spline basis. Determining the number of clusters is still an open question and has been under active ongoing research. This is particularly the case for time course data with a small number of replicates, as it does not allow the application of bootstrapping-based methods, such as the bootstrapped maximum volume measure [6]. Here we fit the CLMM model for several numbers of clusters, including K = 6, K = 7, and K = 8, and chose the number of cluster to be K=8 based on the implicated biology and the empirical examination on cluster separation (Figure 2). As K increased from 6 to 7, a group of 17 genes was separated from a loose cluster and formed a new cluster. According to the clustering estimated in [16] based on the time to the first peak, all 17 genes belong to the G2/M cluster. When K increased from 7 to 8, the major change in gene clustering was that cluster 1 for K = 7 was split into two smaller clusters. See Additional file 1 for the clustering results for K=6 and K=7 and also their comparison with that for K=8. The clusters were labeled to best match the order of the first peak time.

**Figure 3:**
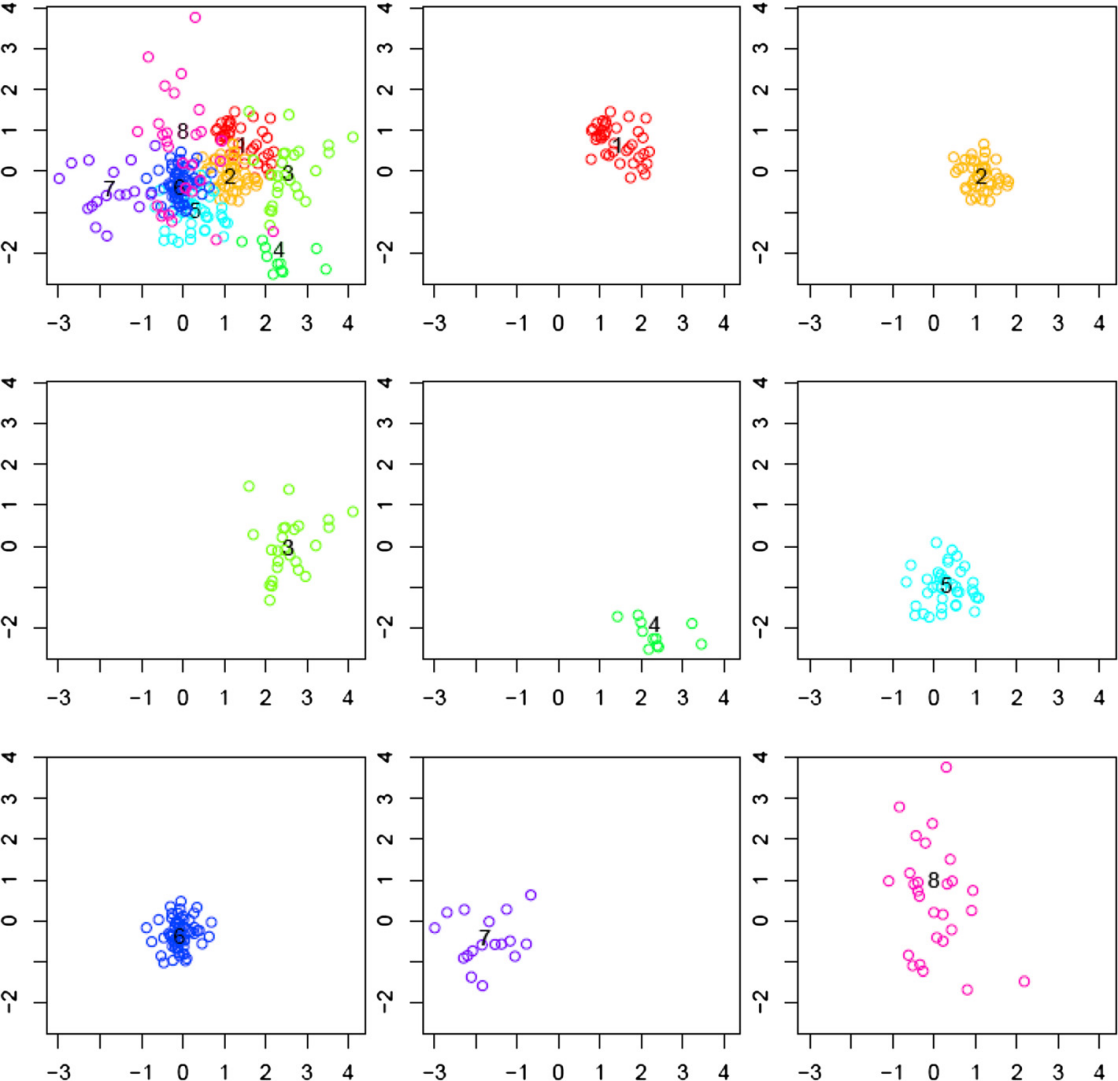
**Scatter plot of the top two eigenvectors for the 256 genes using the WT data.** Panel one plots all genes with genes in the same cluster plotted in the same color. Numbers 1-8 indicate the eight cluster centers. The other eight panels plot genes by cluster.

We did model checking by plotting the model residuals and the Best Linear Unbiased Predictions (BLUPs) (see Additional file 1). Estimated variance of the residuals is fairly constant across time for each of the clusters. Also there is no obvious pattern across time in the residuals, except clusters 3 and 4. Estimated variance of the BLUPs is also reasonably constant across elements of the random effects for each of the clusters. To further explore how the clusters are located to each other and how tight each cluster is, we calculated the eigenvalues for the observed expression data and used the two eigenvectors corresponding to the largest two eigenvalues to display the observed data and the estimated cluster centers with genes in the same cluster highlighted in the same color. The two vectors explain 64.11% of the total variation in the observed data. Figure 3 shows that the clusters partition the samples well, except that cluster 8 overlaps several others and is relatively loose itself.

**Figure 4:**
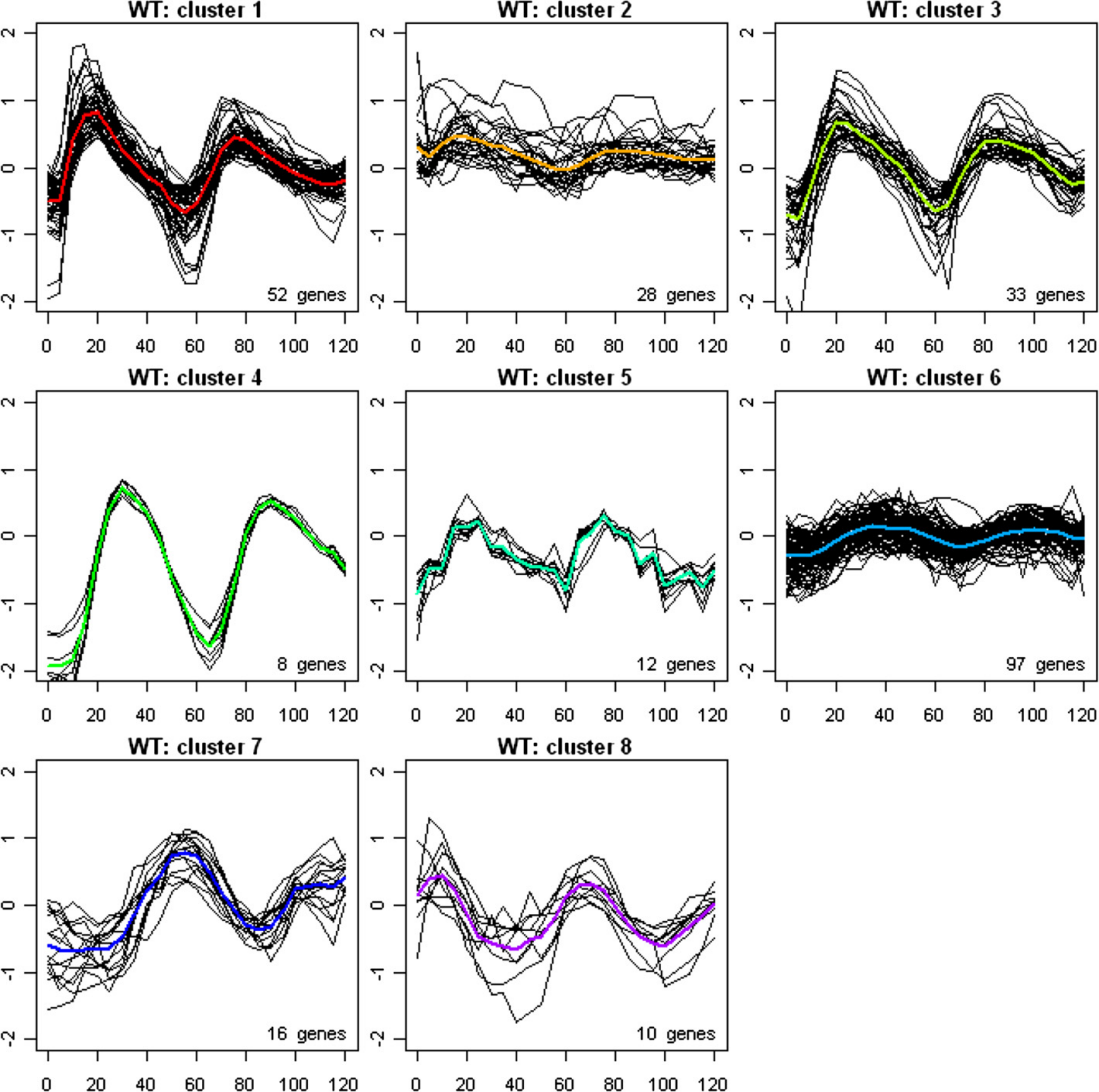
**Clustering of the 256 genes based on K-means clustering using the WT data.** Genes were clustered to eight clusters. Each panel plots the fitted profile (colored line) of one cluster and the observed profiles (black line) of genes in that cluster averaged across the two WT samples *versus* time in minutes. The number of genes in each fitted cluster is labeled at the lower right corner of each panel.

We also clustered the WT data using the K-means method. The resulted clustering is shown in Figure 4, and is compared with the CLMM-based clustering in Table 1. Similar to what we did for the CLMM clusters, the K-means clusters were labeled to best match the order of the first peak time and the CLMM clusters. The two sets of clustering agreed on about 60% genes. K-means, however, lost two CLMM clusters (CLMM cluster 2 and cluster 5), which showed rather homogeneous expression profiles within each cluster. At the same time K-means singled out a new cluster (K-means cluster 5), which was part of CLMM cluster 1: the genes in this new cluster had very bumpy expression profile over the sampling time, which was more likely due to experimental noise than a real biological signal, and they might have been picked up by K-means because of its lack of smoothing across time points.

**Table 1:** Compare the CLMM-based clustering (rows) versus the K-means (KM) based clustering (columns) for the WT data

	**KM 1**	**KM 2**	**KM 3**	**KM 4**	**KM 5**	**KM 6**	**KM 7**	**KM 8**
CLMM WT 1	27				10			
CLMM WT 2	11	10	14			6		
CLMM WT 3	13		10		2			
CLMM WT 4			3	8				
CLMM WT 5			6			32		
CLMM WT 6		9				51		
CLMM WT 7						1	16	
CLMM WT 8	1	9				7		10

#### ***Cluster both WT data and SM data***

**Figure 5:**
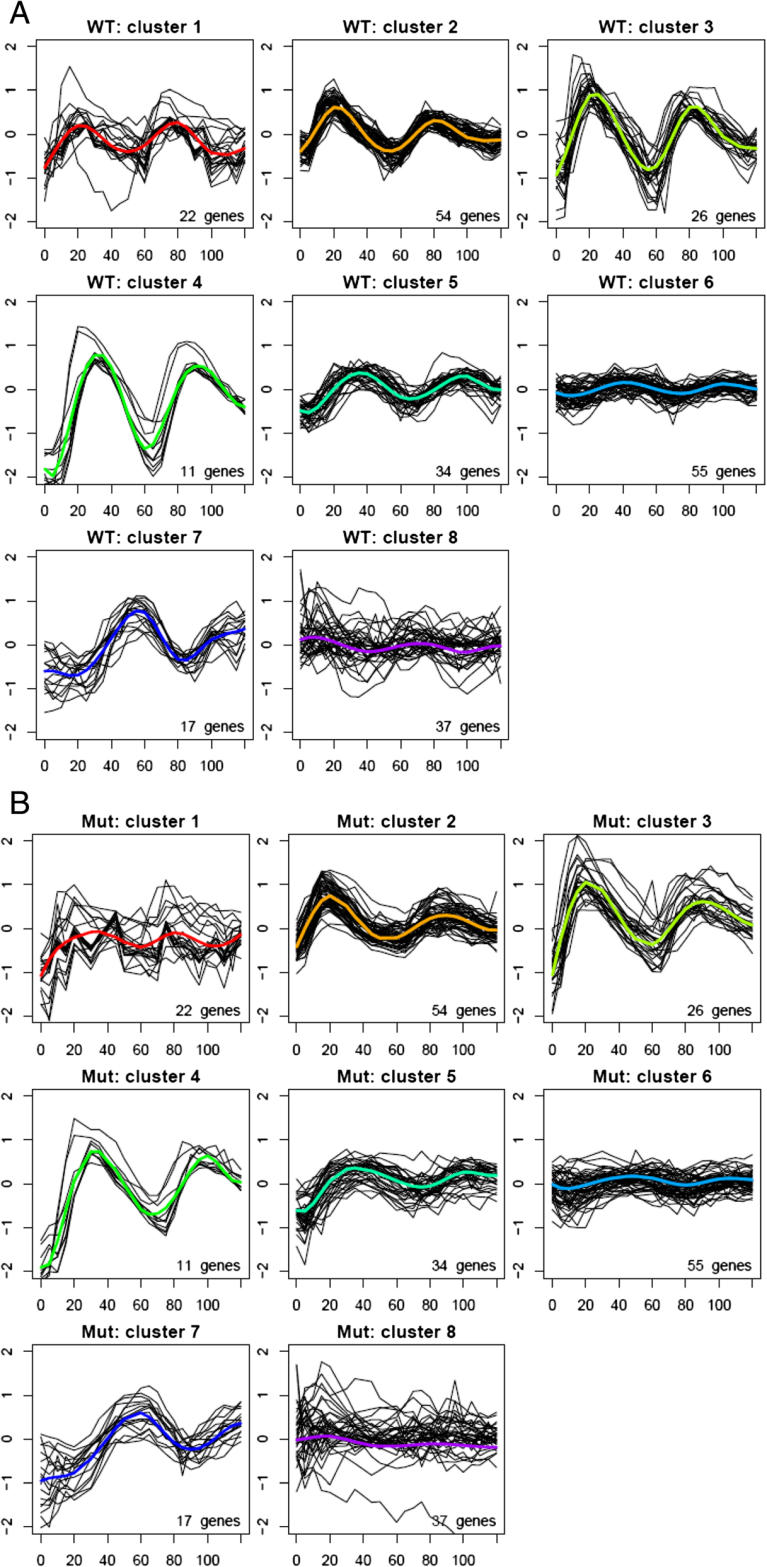
Clustering of the 256 genes based on the CLMM method using both the WT and SM data: (A) WT profiles; (B) SM profiles.

**Figure 6:**
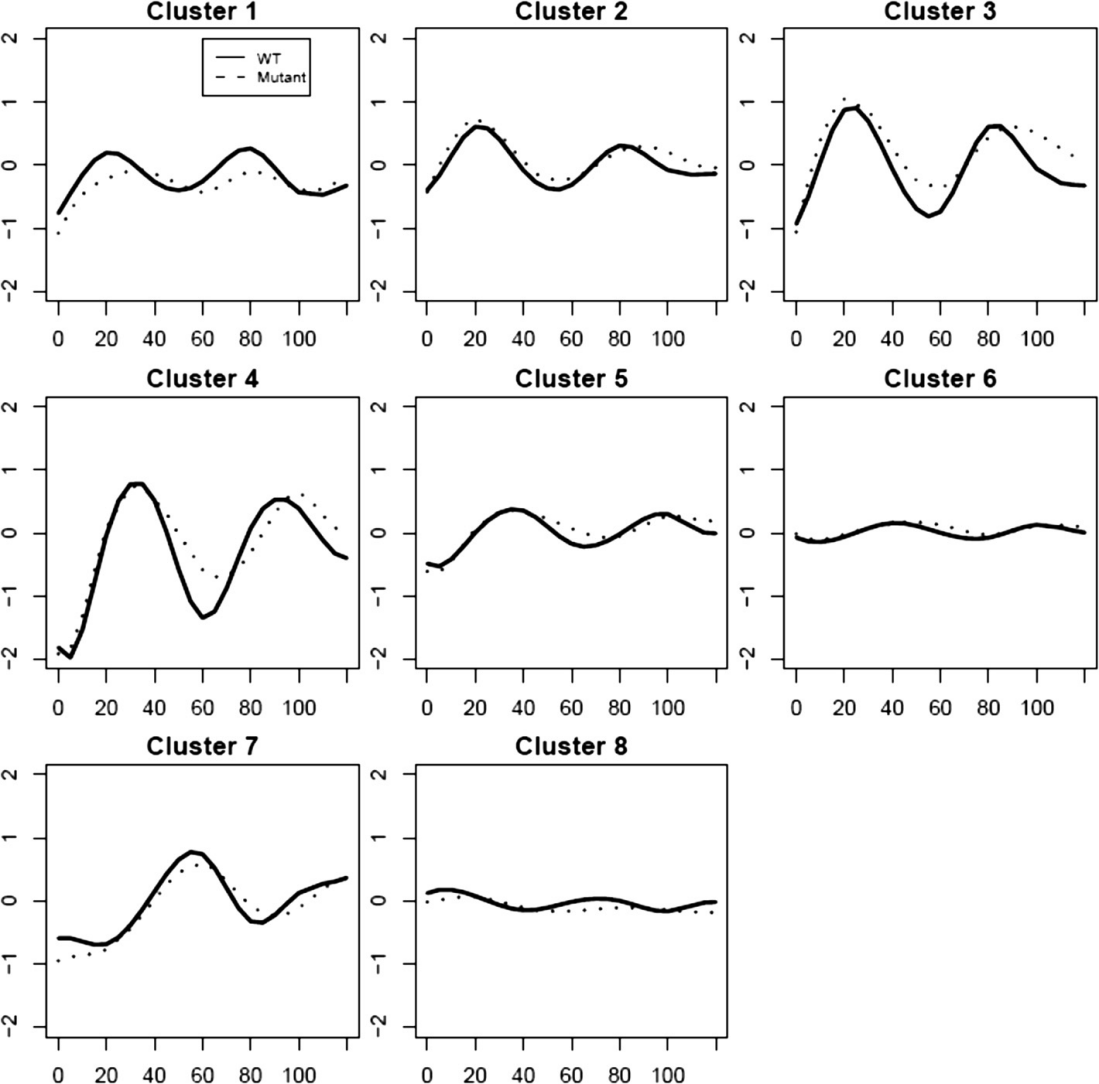
Clustering of the 256 genes based on the CLMM method using both the WT and SM data: fitted WT profiles (solid line); fitted SM profiles (dotted line).

CLMM was also applied to cluster genes using both WT data and SM data. Figure 5 shows the resulted clustering when CLMM is fit with eight clusters. To gain more insights into the underlying biology, the estimated profiles are compared between WT yeast and SM yeast for each cluster (Figure 6). For example, in cluster 1, periodicity is maintained in SM yeast but with a smaller magnitude, which suggests that the mutation may have turned off a repressor for genes in this cluster.

To identify genes whose clustering status is changed by the mutation (that is, ‘differentially clustered’ genes), we compared the clustering using both WT and SM data and that using WT data only (Table 2). This is an empirical approach to identify differentially clustered genes and we would like to further rigorously address this problem in the future. The two clusterings differ mostly in their clusters 1 and 2 and their detailed GO annotation is provided in Additional file 1.

**Table 2:** Compare the CLMM clustering using the WT data (rows) and that using both the WT and SM data (columns)

	**Both 1**	**Both 2**	**Both 3**	**Both 4**	**Both 5**	**Both 6**	**Both 7**	**Both 8**
CLMM WT 1	13	1	3		2			3
CLMM WT 2	21	33						
CLMM WT 3	3		22					1
CLMM WT 4				11				
CLMM WT 5		2			30	2		
CLMM WT 6		1			4	50		
CLMM WT 7							17	
CLMM WT 8		4			2	8		**23**

### Discussion

To summarize, both K-means and CORM are useful tools for clustering genes using expression data. K-means makes no assumption about the relationship between expression levels and sample covariates. It is intuitive and has produced reasonable results in applications [3]. K-means is especially useful to explore the data when no prior knowledge is available on genes’ relationship to covariates. CORM assumes a regression relationship between gene expression and covariate. When the assumption holds, CORM is able to provide more precise clustering and cluster center estimates. Moreover, CORM is capable of naturally handling data with complicated experimental design, for example, longitudinal with replications design, unbalanced time points, and missing data.

Gene clustering for time course data has been under active research over the past decade [18]. A number of other researcher groups have also independently developed gene clustering methods using the mixture of random effects models as the backbone with some variations in the specific modeling and implifications [19-23]. Our results offer further evidence of benefits of the CORM method for time course data over the traditional K-means clustering method. The CORM method is now available as an R package named *CORM* at the R CRAN.

## Competing interests

The authors declare that they have no competing interests.

## Authors’ contributions

LXQ and SGS conceived the study. LB collected the yeast time course data. LXQ carried out the analysis of the yeast data. LXQ, LB, and SGS interpreted the data and wrote the manuscript. All authors read and approved the final manuscript.

## Supplementary Material

Additional file 1 Supplementary materials. Click here for file
