# Supplementary material for: Finding gene clusters for a replicated time course study
Source: BMC Res Notes. 2014 Jan 24;7:60. doi: 10.1186/1756-0500-7-60 (PMC3906880; doi:10.1186/1756-0500-7-60)
Supplement: Additional file 1 — Supplementary materials. [file 1756-0500-7-60-S1.doc]

**Supplementary Materials**
